# Diagnosis and treatment of paragangliomas and pheochromocytomas: a survey from the Italian Association for Neuroendocrine Tumors

**DOI:** 10.3389/fendo.2025.1687570

**Published:** 2025-09-29

**Authors:** Erica Pietroluongo, Antonella Lucia Marretta, Mauro Cives, Elisa Andrini, Leonardo Della Sala, Elisabetta Dell’Unto, Alice Carli, Nevena Mikovic, Ilaria Mascagni, Francesco Panzuto, Salvatore Tafuto

**Affiliations:** ^1^ Department of Clinical Medicine and Surgery, University of Naples Federico II, Naples, Italy; ^2^ Melanoma, Cancer Immunotherapy and Development Therapeutics Unit, Istituto Nazionale Tumori IRCCS Fondazione Giovanni Pascale, Naples, Italy; ^3^ Interdisciplinary Department of Medicine, University of Bari “Aldo Moro”, Bari, Italy; ^4^ Department of Medical and Surgical Sciences (DIMEC), Alma Mater Studiorum - Università di Bologna, Bologna, Italy; ^5^ Endocrinology Unit, Department of Internal Medicine and Medical Specialties, School of Medical and Pharmaceutical Sciences, University of Genova, Genova, Italy; ^6^ Department of Medical-Surgical Sciences and Translational Medicine, Digestive Disease Unit, Sant’Andrea University Hospital, European Neuroendocrine Tumor Society (ENETS) Center of Excellence, Sapienza University of Rome, Rome, Italy; ^7^ Radiology Unit, Diagnostic and Public Health Department, University of Verona, Policlinico GB Rossi, Verona, Italy; ^8^ Endocrinology Unit, Department of Clinical and Molecular Medicine, European Neuroendocrine Tumor Society (ENETS) Center of Excellence, Sant’Andrea Hospital, Sapienza University of Rome, Rome, Italy; ^9^ Department of Biomedical Sciences, Humanitas University, Milan, Italy; ^10^ Department of Sarcoma and Rare Tumors, Istituto Nazionale Tumori I.R.C.C.S. European Neuroendocrine Tumor Society (ENETS) Center of Excellence Fondazione “G.Pascale”, Naples, Italy

**Keywords:** pheochromocytomas, paragangliomas, neuroendocrine neoplasms, genetic counseling, imaging, treatment

## Abstract

**Background:**

Pheochromocytomas (PCCs) and paragangliomas (PPGLs) are rare neuroendocrine neoplasms (NENs) with heterogeneous clinical presentations. Given the rarity of PCCs/PPGLs and the paucity of high-level evidence, therapeutic decisions and treatment sequences vary across institutions. This survey explored current diagnostic practices and treatment patterns among Italian healthcare professionals (HCPs) dedicated to NENs.

**Methods:**

An online survey was conducted among Italian HCPs, members of the Italian Association for Neuroendocrine Tumors (ITANET). The survey included 33 questions covering diagnosis, genetic counseling, imaging, and treatment approaches. Responses were collected from December 15, 2023, to May 30, 2024, and analyzed using descriptive statistics to identify trends in clinical practice.

**Results:**

We recorded 80/355 responses from invited HCPs (response rate: 23%). Most HCPs (90%) referred all PCC/PPGL patients for genetic counseling, with 71% adopting gene panels for syndromes like VHL, MEN2, and familial PPGLs. Functional imaging preferences included ^68^Ga-DOTA-peptide PET/CT (38%), ^18^F-DOPA PET/CT (26%), and ^123^I-MIBG scintigraphy (19%). First-line systemic treatments favored somatostatin analogs (39%), clinical trial enrollment (19%), and CVD chemotherapy (15%). Radioligand therapy (RLT) emerged as the preferred second-line treatment (49%). Overall, RLT was perceived as the most effective treatment for achieving objective responses, durable responses, and improving health-related quality of life.

**Conclusions:**

Clinical wisdom rather than formal evidence and guidelines recommendations appears to guide the management of PCC/PPGLs among Italian HCPs. International, multi-institutional clinical trials designed to take into account the rarity of PCCs/PPGLs are needed to generate high-level evidence and provide guidance for standard clinical practice.

## Introduction

Pheochromocytomas (PCCs) and paragangliomas (PPGLs) are rare neuroendocrine neoplasms (NENs) originating from the adrenal medulla and extra-adrenal paraganglia respectively (1). According to their anatomical origin, PPGLs can be subdivided into sympathetic or parasympathetic PPGLs. While PCCs and sympathetic PPGLs may produce and secrete catecholamines and other bioactive substances, parasympathetic PPGLs tend to be non-functioning. Taken together, PCCs and PPGLs have an incidence of approximately 0.6 cases per 100,000 persons/year and tend to arise after the fourth decade of life (2). In patients with functioning tumors, presenting symptoms include palpitations, hypertension, sweating, and headaches.

A substantial proportion of PCCs/PPGLs arise in the context of hereditary syndromes including Von-Hippel Lindau (VHL) disease, Multiple Endocrine Neoplasia type 2 (MEN2) syndrome, neurofibromatosis and familial PPGLs syndrome (3). Hereditary PCCs/PPGLs tend to have distinctive clinical features as compared with sporadic neoplasms. Regardless of their hereditary or sporadic nature, PCCs/PPGLs can be molecularly subdivided into three major oncogenic clusters, termed pseudo-hypoxic cluster (cluster 1A and 1B), kinase signaling pathway cluster (cluster 2) and WNT signaling cluster (cluster 3) (4). Such molecular classification has both prognostic and predictive ability (5).

Functional imaging has a defined place in the diagnostic work-up and management of PCCs/PPGLs. Among the most effective imaging modalities for detecting PCCs and PPGLs there is somatostatin receptor (SSTR) PET-based imaging or ^131^I-meta-iodobenzylguanidine (MIBG) SPECT/CT (6). Various radiotracers including DOTA-Tyr3-octreotate (DOTATATE), DOTA-Tyr3-octreotide (DOTATOC), and DOTA-Nal3-octreotide (DOTANOC) are currently employed for SSTR imaging in patients with PCC/PPGLs, and results in terms of diagnostic performance appear comparable (7).

The treatment landscape of metastatic PCCs/PPGLs has recently expanded. While alpha and beta blockers as well as catecholamine synthesis inhibitors represent the mainstay for the palliation of secretory symptoms, somatostatin analogs, tyrosine kinase inhibitors (i.e., sunitinib), chemotherapy, radiopharmaceutical therapies involving radioiodine or SSTR-targeting radiopeptides and locoregional therapies including cytoreductive surgery, external beam radiotherapy, arterial embolization and cryotherapy are potential options to control tumor growth (8). Among chemotherapy regimens, the combination cyclophosphamide-vincristine-dacarbazine (CVD) and the monotherapy with temozolomide have shown evidence of antitumor activity against PCCs/PPGLs (9, 10). Overall, the activity of temozolomide appears higher in patients affected by PPGLs carrying mutations in the *SDHB* gene, possibly as result of increased frequency of *MGMT* promoter hypermethylation (11).

No universally accepted optimal treatment sequences exist for the management of PCCs/PPGLs. Treatment is usually individualized based on patient characteristics (i.e., age, co-morbidities, etc.) and tumor-related factors (i.e., secretory characteristics, mutational/molecular background, extent of disease, pace of growth) (12). Goals of treatment include palliation of hormonal symptoms, control of cardiovascular complications, amelioration of health-related quality of life and prolongation of survival.

Given the rarity of PCCs/PPGLs, the inherent challenges in conducting large-scale clinical trials and the consequent paucity of high-level evidence in the field, therapeutic decisions and treatment sequence planning often vary across institutions, being influenced by the specific expertise of the center and the experience of the treating physician. To gain a comprehensive understanding of the perspectives of Italian physicians treating PCCs/PPGLs, we conducted an online survey among ITANET (Italian Association for Neuroendocrine Tumors) members to explore current diagnostic practices and treatment patterns. While most ITANET members are familiar with the treatment of PCCs/PPGLs, almost all Italian physicians dealing with PCCs/PPGLs are members of ITANET, thus guaranteeing the representativeness of the survey sample at national level.

## Patients and methods

### Survey design

An online survey was conducted among Italian healthcare professionals (HCPs) dedicated to NENs. The survey was designed by members of ITANET (ST, EP, ALM) during the ITANEXT Spring Meeting (an annual meeting fostering multidisciplinary collaboration between young medical doctors committed to NENs in Italy). The survey (**Supplementary Material**) covered various aspects of diagnosis, treatment and multidisciplinary care of PCCs/PPGLs and was aimed at capturing current practices/approaches across different institutions distributed in various Italian regions. The survey invitation was distributed to all ITANET members through the Google Forms platform (https://www.google.com/forms/about/). Participation was voluntary and implied consent to the subsequent use of the data, that were processed anonymously. Responses were collected from December 15, 2023 to May 30, 2024. The study was conducted in compliance with the Declaration of Helsinki, and did not require ethical committee approval due to its non-interventional nature.

### Survey measures

The survey collected sociodemographic data of respondents, context variables, and several critical outcomes of interest related to the diagnosis and management of PCCs/PPGLs. These outcomes included clinical management practices, genetic counseling, adoption of advanced imaging techniques and treatment choices/preferences. The survey was in Italian language and consisted of 33 question items, including yes/no, single- or multiple-choice, and open-ended questions.

### Statistical analysis

Descriptive statistics was primarily used for reporting survey results. Percentages and frequency distributions were used to summarize the responses across different categories. The analysis focused on identifying trends in clinical practice, with no inferential statistical tests performed due to the exploratory nature of the survey.

## Results

### Respondents and respondents’ institutions

**Figure 1:**
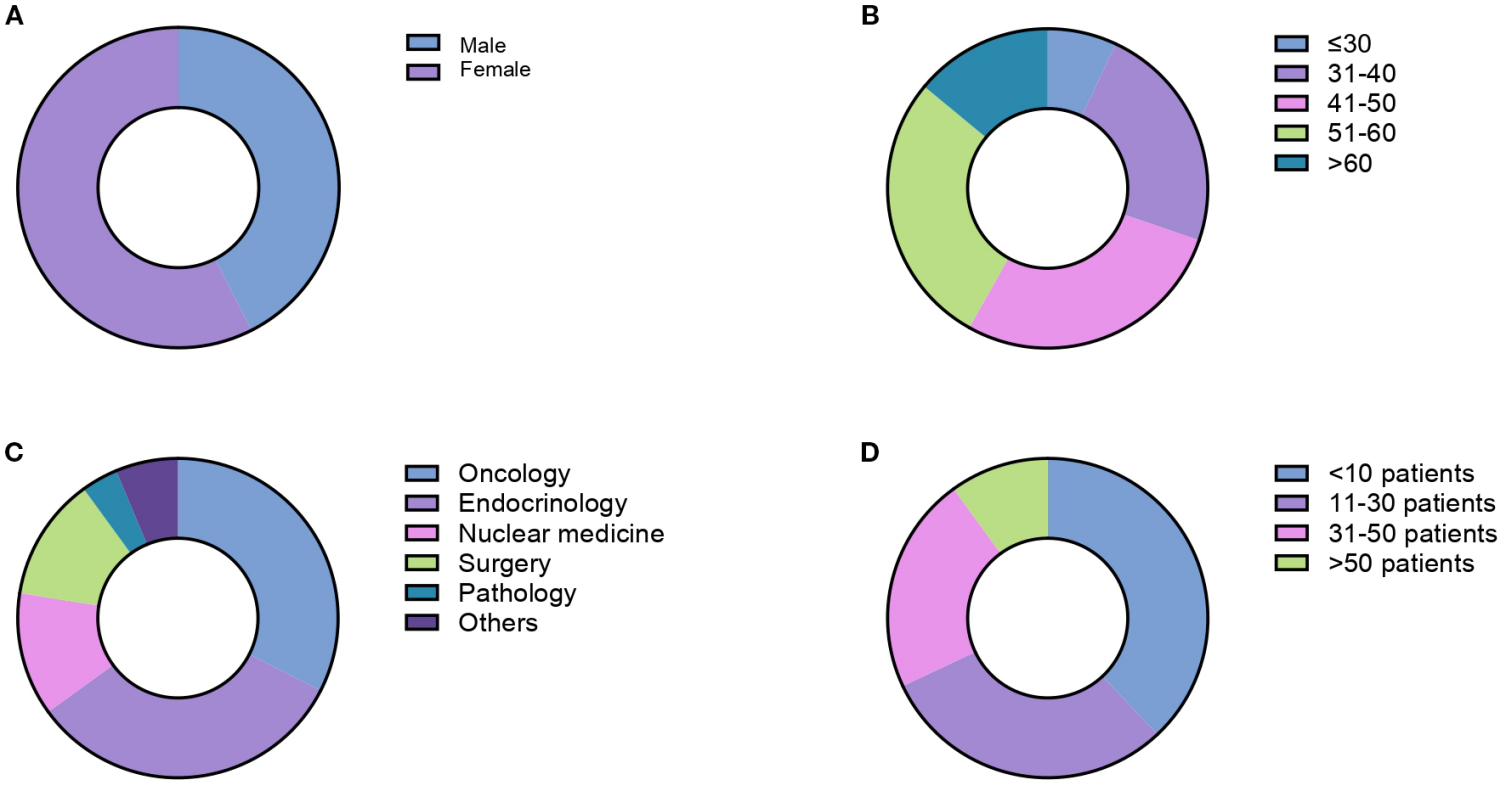
Demographic and professional characteristics of survey respondents including gender distribution **(A)**, age distribution **(B)**, medical specialties **(C)** and number of PCC/PGL cases diagnosed in the last 10 years **(D)**.

Three hundred fifty-five HCPs were invited to complete the survey. Eighty responded, resulting in a response rate of 23%. The HCPs were female in 58% of cases (**Figure 1A**). Regarding the age distribution, 7% were under 30, 23% aged 31-40, 28% aged 41-50, 28% aged 51-60, and 14% over 60 (**Figure 1B**). The specialties of the respondents included oncology (33%), endocrinology (33%), general surgery (12%), nuclear medicine (12%), pathology (4%), and other specialties (6%) (**Figure 1C**). HCPs had a medical experience of 2–5 years in 34% of cases, 6–10 years in 30% of cases, 11–20 years in 16% of cases and more than 20 years in 20% of cases.

Respondents’ institutions covered a broad geographic area in Italy, being located in 20/20 different regions. Most institutions were research institutions (i.e., comprehensive cancer centers, tertiary research hospitals, etc.; 48%), followed by university hospitals (20%), public hospitals (15%), private hospitals or other facilities (17%). Overall, 36% of the respondents worked in European Neuroendocrine Tumor Society (ENETS) Centers of Excellence. The number of PCCs/PPGLs diagnosed at respondents’ institutions over the past 10 years was <10 in 38% of cases, 11–30 in 30% of cases, 31–50 in 22% of cases, >50 in 10% of cases (**Figure 1D**). All PCC/PPGL cases were discussed in multidisciplinary tumor boards in 82% of cases. Overall, 32% of respondents referred PCC/PPGL patients to ENETS Center of Excellence or consulted with other institutions having a specific expertise in PCC/PPGLs.

### Pathological reporting, genetic counseling, functional imaging

Pathology reports of PCCs/PPGLs were consistent with the WHO recommendations (13) in 30% of cases. According to institutional practices, pathology reports included information on Ki-67, S100, GATA3, SDHB, SDHA, MAX, 2SC, PSS and/or GAPP score in 30% of cases. The same information with the exclusion of PSS and/or GAPP score were available in the pathology reports of 25% of respondents. Another 16% of HCPs indicated that their pathology reports excluded only the GAPP score. Twenty-nine percent of HCPs declined to provide an answer.

The vast majority of respondents (90%) referred all patients with PCCs/PPGLs for genetic consult. In this context, most HCPs (71%) adopted gene panels investigating genetic syndromes including VHL disease, MEN2 and familial PPGLs. Five percent of respondents investigated the presence of mutations leading to VHL disease, MEN2 and neurofibromatosis. Approximately 8% evaluated only the presence of RET mutations, while 2% mentioned conducting molecular analysis based on clinical suspicion. Patients were referred to the geneticist without a clear idea of the possible underlying genetic syndrome by 4% of respondents.

Among functional imaging modalities, ^68^Ga-DOTA-peptide PET/CT was the preferred technique (38%), followed by ^18^F-DOPA PET/CT (26%), scintigraphy with ^123^I-MIBG (19%) and FDG-PET/CT (8%). Approximately 5% of respondents indicated that they used a combination of ^68^Ga-DOTA-peptide and ^18^F-DOPA PET/CT as preferred functional imaging modality. Approximately 4% of HCPs refrained from responding.

### First-line treatment preferences

**Figure 2:**
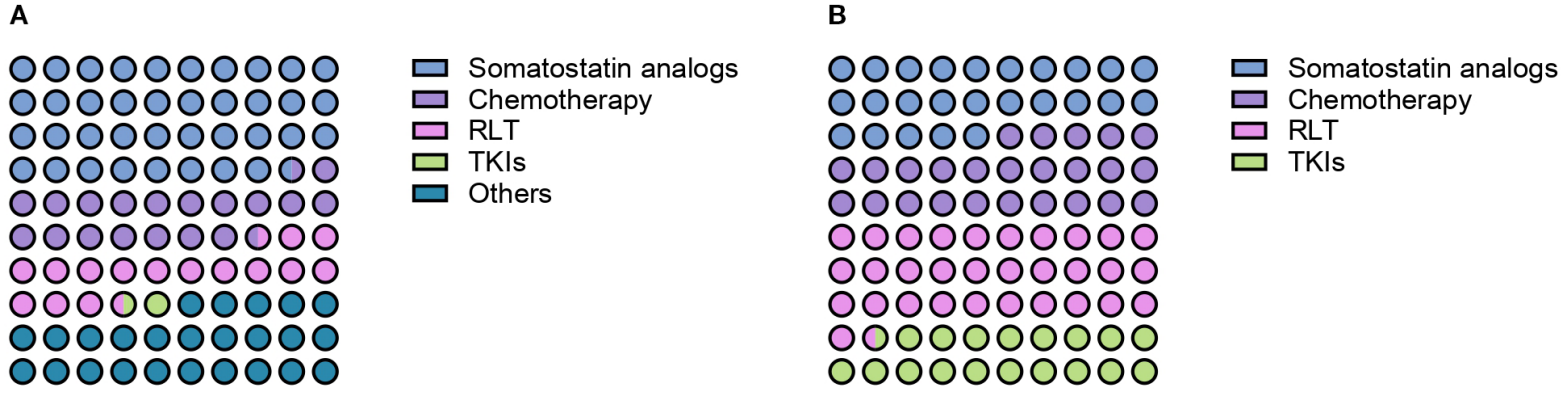
Preferred first- **(A)** and second-line therapeutic options **(B)** for patients with advanced PCCs/PPGLs according to survey respondents.

When interrogated about preferences for the first-line systemic treatment of advanced PCCs/PPGLs, HCPs indicated somatostatin analogs as the preferred option (39%). Enrollment in clinical trials, CVD chemotherapy, radiopharmaceutical therapy and temozolomide were preferred by 19%, 15%, 1% and 1% of respondents respectively. Twenty-five percent of HCPs opted for different management strategies, including active surveillance (**Figure 2A**).

**Figure 3:**
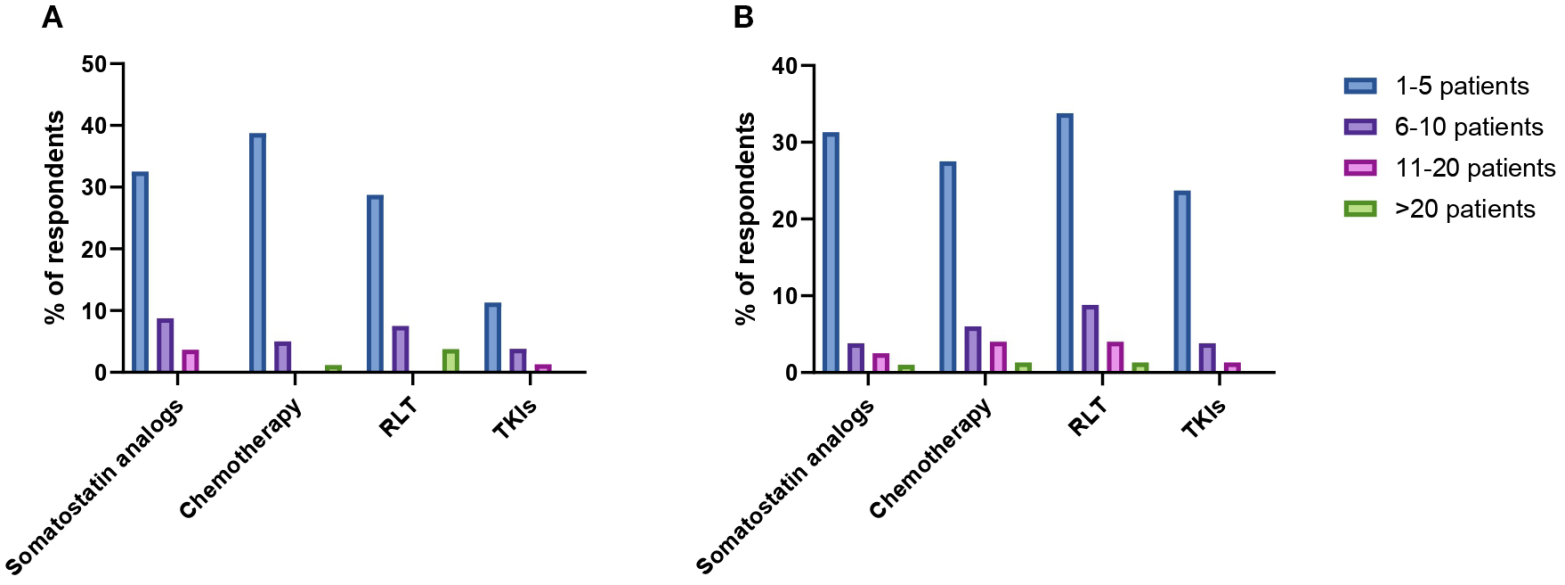
Reported number of patients with PCCs/PPGLs treated with somatostatin analogs, chemotherapy, radioligand therapy (RLT) and tyrosine-kinase inhibitors (TKIs) in the first- **(A)** and second-line treatment setting **(B)**.

When specifically asked about the number of patients with PCCs/PPGLs treated with frontline somatostatin analogs in the past 10 years, treatment of 0, 1–5 patients, 6–10 patients, 11–20 patients and more than 20 patients was reported by 50%, 33%, 9%, 4% and 4% of respondents respectively. Treatment of 1–5 patients, 11–20 patients and more than 20 patients with first-line CVD chemotherapy was reported by 39%, 5% and 1% of practitioners. Fifty-five percent of HCPs declared that they did not treat any patients with CVD chemotherapy in the past decade. The frontline use of radioligand therapy (RLT) was rather limited, with 29% of HCPs reporting the treatment of 1–5 patients, 8% of them having treated 6–10 patients and 4% more than 20 patients in the past decade. TKIs were the least used first-line option, with 11% of respondents reporting the treatment of 1–5 patients, 4% of 6–10 patients and 1% of 11–20 patients. **Figure 3A** provides an overview of the number of patients treated with each distinct treatment modality in the first-line setting in the past 10 years.

### Second-line treatment preferences

HCPs were next interrogated regarding their treatment preferences beyond first-line therapy. Each HCP could provide more than one answer in terms of preferred option. RLT was the preferred second-line treatment option according to the 49% of respondents (**Figure 2B**). Thirty-four percent of HCPs reported the use of second-line RLT in 1–5 patients with PCCs/PPGLs in the past decade, whereas treatment of 6–10 patients, 11–20 patients and more than 20 patients was reported by 9%, 4% and 2% of respondents respectively. The most widely employed radiopharmaceuticals were ^131^I-MIBG (21%) and ^177^Lu-DOTA-peptide (20%), followed by ^90^Y-DOTA-peptide (13%) and combinations of ^90^Y- and ^177^Lu-DOTApeptides (6%). Thirty-nine percent of respondents reported the use of somatostatin analogs as second line therapeutic option for patients with metastatic PCCs/PPGLs over the past ten years. Overall, 31% of them reported the treatment of 1–5 patients, 4% of 6–10 patients, 3% of 11–20 patients and 1% of more than 20 patients. A similar proportion of HCPs (39%) indicated that used chemotherapy in the second-line setting. Treatment of 1–5 patients, 6–10 patients, 11–20 patients and more than 20 patients was reported by 28%, 6%, 4% and 1% of respondents respectively. Among chemotherapeutic regimens to be employed in the second-line setting, HCPs mentioned temozolomide monochemotherapy (58%), CVD (21%) and CAPTEM (21%). TKIs were indicated as preferred second-line option by 29% of respondents. In the past decade, 24% of HCPs treated 1–5 patients with TKIs, while 6–10 patients and 11–20 patients were treated with TKIs by 4% and 1% of practitioners respectively. Enrollment in clinical trials was indicated as a suitable strategy by 26% of respondents. Numbers of patients treated with different treatment modalities in the second-line setting are summarized in **Figure 3B**.

### Objective responses, duration of response, health-related quality of life

**Figure 4:**
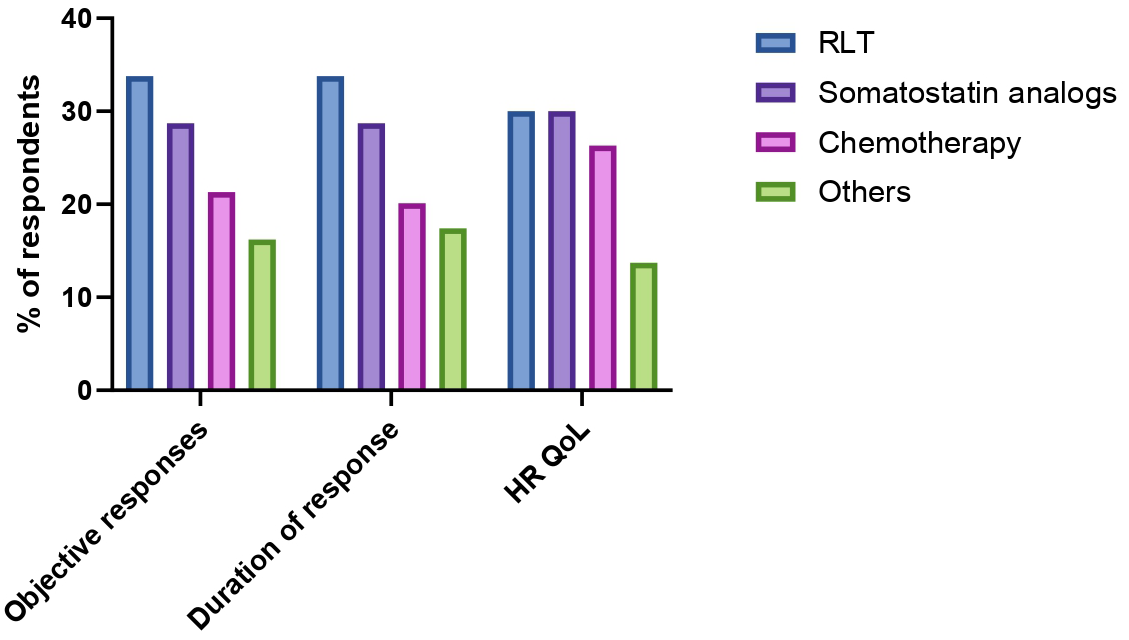
Perceived safety and efficacy of radioligand therapy (RLT), somatostatin analogs, chemotherapy and other treatments (irrespective of treatment line) according to key domains (possibility to induce tumor shrinkage, duration of response, health-related quality of life (HR QoL).

The perceived efficacy of therapies against PCCs/PPGLs was investigated through the exploration of three different domains: ability to induce tumor shrinkage, ability to induce durable responses, ability to improve health-related quality of life. RLT was identified as the most effective treatment for achieving objective responses (34% of responses), followed by somatostatin analogs (29% of responses) and chemotherapy (21% of responses). Among chemotherapeutic regimens, CVD and temozolomide were selected as preferred option when tumor shrinkage was the goal by 13% and 9% of respondents respectively. Sixteen percent of HCPs identified other treatments (including investigational therapies) as the most effective in inducing objective responses. Similar results were obtained when HCPs were interrogated on the perceived durability of treatment responses. RLT was indeed identified as the option most likely to guarantee long-lasting responses by 34% of respondents, followed by somatostatin analogs (29% of responses), chemotherapy (20% of responses) and other treatments (17% of responses). Durability of responses upon treatment with CVD or temozolomide chemotherapy was perceived as similar. When the focus of treatment efficacy was the improvement of health-related quality of life of patients with advanced PCCs/PPGLs, RLT and somatostatin analogs were equally favored as preferred options (30% of responses each), followed by chemotherapy with CVD (21%). Temozolomide and other treatments (including the combination RLT/somatostatin analogs) were identified as the treatments most likely to improve the health-related quality of life by 5% and 14% of respondents respectively. HCP’s perceptions on distinct treatment efficacy measures are summarized in **Figure 4**.

## Discussion

Our work provides a picture of the current patterns of management of advanced PCCs/PPGLs in Italy. Although substantial variability exists across institutions, clear trends emerged in terms of treatment preferences and coordinated multidisciplinary care.

One of the most striking areas of almost complete consensus in our survey was the need for genetic counseling in all patients diagnosed with PCCs/PPGLs, in accordance with the most recent ESMO-EURACAN recommendations (14). PCCs and PPGLs are the most hereditary tumors known, and more than 20 genetic driver variants lacking a clear genotype-phenotype correlation have been identified so far (15). While 90% of the HCPs interviewed by our survey reported to routinely refer patients diagnosed with PCCs/PPGLs to genetic consultation, inter-institutional discrepancies emerged in terms of number and type of genes subjected to sequencing. Development of comprehensive gene panels specifically designed for the diagnosis of genetic conditions associated with PCCs/PPGLs might improve the recognition of hereditary PCC/PPGLs. Centralization of genetic testing (on a national or regional level) for such rare malignancies might also contribute to costs reduction and uniformity in reporting.

Although advanced nuclear medicine imaging techniques were uniformly integrated in the management of patients with PCCs/PPGLs according to our survey results, no clear preferences towards ^68^Ga-DOTA-peptide PET/CT, ^18^F-DOPA PET/CT, ^18^F-FDG-PET/CT or ^123^I-MIBG scintigraphy emerged. While this is consistent with guidelines (14), it also highlights the incomplete clinical implementation of recent evidence showing the superiority of SSTR imaging versus other functional imaging modalities, particularly in the context of PPGLs (16–18).

Another area of high consensus among survey respondents was the need for multidisciplinary, expert management of patients with PCCs/PPGLs. Indeed, more than 80% of the interviewed HCPs indicated that all cases of PCCs/PPGLs were routinely discussed in the context of dedicated multidisciplinary tumor boards, and approximately one third of respondents reported to refer patients to ENETS Centers of Excellence or other centers of expertise. These figures should be interpreted in light of the characteristics of the interviewed HCPs (all partly or fully dedicated to NENs), and underscore the concept that the management of PCCs/PPGLs may benefit of expert opinions even when the treating physician deals with high volumes of other NENs. The implementation at a national level of an expert network accessible for multidisciplinary discussion of PCC/PPGL cases might further improve patients’ outcomes, pushing the boundaries of the current institution-based multidisciplinary care.

Clear trends in first- and second-line therapeutic preferences emerged from our survey. Approximately 40% of respondents indicated somatostatin analogs as preferred frontline therapeutic option in patients with advanced PCC/PPGLs. While no high-level evidence exists to support such a preference, this treatment pattern underscores the established role of somatostatin analogs in “agnostically” targeting SSTR-expressing tumors, particularly within a community of physicians used to manage NENs. Whether somatostatin analogs significantly delay tumor progression as compared with active surveillance in patients with PCCs/PPGLs remains a conundrum. The LAMPARA trial, a phase 2 study investigating lanreotide in patients with advanced PCCs/PPGLs, (NCT03946527) will hopefully shed light on this aspect.

Notably, approximately one fifth of survey respondents considered enrollment in clinical trials as the preferred option for the first-line treatment of patients with advanced PCCs/PPGLs. This reflects the ongoing interest in investigating new therapeutic modalities in neoplasms once considered too rare to be formally studied in the context of clinical trials. At present, a clinical trial of the HIF-2alpha inhibitor belzutifan is actively enrolling patients with treatment-naïve PCCs/PPGLs across multiple institutions in Italy (NCT04924075).

SSTR targeting through either cold or radiolabeled somatostatin analogs emerged as the preferred option for the second-line treatment of patients with PCCs/PPGLs. In particular, RLT was considered as potential second-line treatment strategy by approximately half of respondents, with the majority of HCPs preferring SSTR-targeting radiopharmaceuticals over radioiodine. Notably, while over the last few decades SSTR-based RLT has been administered in Italy using in-house produced radiolabeled somatostatin analogs, current regulations hamper such an approach. The design of clinical trials of SSTR-based RLT specifically focusing on patients with advanced PCCs/PPGLs becomes therefore crucial to allow the treatment of these patients as well as to gather formal evidence of therapeutic activity possibly leading to regulatory approval. This aspect appears of paramount importance, also in light of the HCPs’ perceptions gathered in this survey defining RLT as the treatment most likely to induce both objective responses, durable responses and health-related quality of life preservation in patients with PCCs/PPGLs.

Chemotherapy and TKIs were indicated as preferred second-line treatments by approximately 40% and 30% of survey respondents. Notably, temozolomide (either alone, in combination with capecitabine or administered through metronomic schedules) was the most frequently used agent, highlighting a shift from more intensive regimens (i.e., CVD or CDD) to more tolerable ones. The use of TKIs was rather limited according to survey results, especially in consideration of the fact that formal evidence of sunitinib efficacy exists based on the results of the FIRSTMAPP trial (19). Lack of regulatory approval and need for institutional authorization for reimbursement purposes may have an impact on TKI use in Italy.

Taken together, our survey results show that a certain degree of discrepancy exists between guidelines recommendations (13) and clinical practice. This gap is particularly noteworthy in consideration of the fact that most survey respondents treat large numbers of NENs, and inconsistencies cannot therefore derive from lack of knowledge or clinical expertise, but are rather related to the absence of high-level evidence in the field. Rationally conducted clinical research, possibly employing innovative trial designs accounting for the rarity of PCCs/PPGLs, will have a key role in reshaping the management of such malignancies in the next decade.

Our work has several limitations. First, the response rate to the online survey was as low as 23%. Although this figure is not uncommon among surveys targeting clinical oncologists, low response rates can introduce several types of bias including non-response bias and selection bias. Non-response bias may skew the results if the level of experience, institutional affiliations and clinical practice of non-respondents systematically differ from those who responded. Selection bias may also confound our findings, as HCPs with a specific interest in PCCs/PPGLs, or with more time or motivation to engage in surveys may have been more likely to participate. As result of both non-response bias and selection bias, individuals who responded to our survey might not be fully representative of all Italian HCPs treating PCCs/PPGLs, thus potentially limiting the external validity of our results. Over- or under-representation of specific demographics or professional groups among our survey respondents may have also affected the generalizability of our findings. Specifically, an over-representation of HCPs working in ENETS Centers of Excellence may be identified among our survey respondents. ENETS Centers of Excellence typically have more specialized expertise and resources for managing PCCs/PPGLs, which may not reflect the practices and challenges faced in non-specialized settings. Lastly, recall bias and survivorship bias may have affected some of the responses of surveyed HCPs, limiting their accuracy. This is particularly true for questions requiring retrospective reporting of clinical decisions or patient outcomes.

## Conclusions

The first-line treatment of PCCs/PPGLs in Italy mainly consists of somatostatin analogs, whereas RLT and chemotherapy represent preferred second-line treatment choices. According to HCPs’ perceptions, RLT is characterized by the highest potential of inducing objective responses, the highest capability of determining durable responses as well as the highest probability of improving health-related quality of life in patients with advanced PCCs/PPGLs. Such perceptions rely on clinical wisdom rather than on formal studies and high-level evidence. While clinical trials investigating new therapies as well as treatment sequences for patients with advanced PCCs/PPGLs are urgently needed, the development of new national and international guidelines is key to incorporate newly established evidence into standard clinical practice, thereby optimizing patients’ management. Genetic counseling and multidisciplinary management in expert centers remain pivotal elements in the management of PCCs/PPGLs, and may benefit of nation-level centralization.

## Data Availability

The raw data supporting the conclusions of this article will be made available by the authors, without undue reservation.
