# Supplementary material for: Diagnosis and treatment of paragangliomas and pheochromocytomas: a survey from the Italian Association for Neuroendocrine Tumors
Source: Front Endocrinol (Lausanne). 2025 Sep 29;16:1687570. doi: 10.3389/fendo.2025.1687570 (PMC12515617; doi:10.3389/fendo.2025.1687570)
Supplement: Supplementary file 1 [file DataSheet1.docx]

**Diagnosis and Treatment of Paraganglioma and Pheochromocytomas: A Survey from the Italian Association for Neuroendocrine Tumors (IT.A.NET)**

IT.A.NET is the Italian Association for Neuroendocrine Tumors, founded in 2010 with the aim of promoting both basic and clinical research for the management of patients affected by neuroendocrine tumors.

Pheochromocytomas (PCC) and paragangliomas (PGL) are tumors derived from the adrenal medulla and extra-adrenal paraganglia, respectively, with an incidence of approximately 0.6 cases per 100,000 person-years. Data regarding the efficacy of systemic treatments for these rare neoplasms are derived from retrospective studies. The recommendations in published guidelines are expert consensus-based and stem from the limited available data, integrated with the experience of involved clinicians and knowledge extrapolated from other neuroendocrine neoplasms.

The analysis of this anonymized survey's results will be useful for the publication of a report to better understand the optimal diagnostic and clinical management strategies for paragangliomas and pheochromocytomas (PPGL) in major Italian centers, considering that, to date, no widely accepted standard sequential treatments exist.

**Region of Affiliation**

(free text field)

**First Name, Last Name, and email address**

(free text field)

**Gender**

- ☐ Male
- ☐ Female
- ☐ Not Specified

**Age**

- ☐ <30
- ☐ 31-40
- ☐ 41-50
- ☐ 51-60
- ☐ >60

**Is your center an ENETS Center of Excellence?**

- ☐ Yes
- ☐ No

**What type of institution do you work in?**

- ☐ University Hospital
- ☐ IRCCS (Institute for Research, Hospitalization, and Healthcare)
- ☐ Public Hospital
- ☐ Private Hospital
- ☐ Private Practice
- ☐ Other (please specify)

**What is your medical specialty?**

- ☐ Oncologist
- ☐ Endocrinologist
- ☐ Surgeon
- ☐ Nuclear Medicine Physician
- ☐ Pathologist
- ☐ Gastroenterologist
- ☐ Other (please specify)

**How many years of experience do you have in your discipline, particularly in the field of rare tumors?**

- ☐ <2 years
- ☐ 2-5 years
- ☐ 6-10 years
- ☐ 11-20 years
- ☐ >20 years

**How many patients diagnosed with paraganglioma and/or pheochromocytoma have been treated at your institution in the past 10 years?**

- ☐ <10 patients
- ☐ 11-30 patients
- ☐ 30-50 patients
- ☐ 51-100 patients
- ☐ >100 patients

**How many patients with paraganglioma and/or pheochromocytoma have you personally managed at your institution in the last 10 years?**

- ☐ 0
- ☐ 1-5
- ☐ 5-10
- ☐ 11-30
- ☐ >30

**Are cases of paraganglioma and pheochromocytoma discussed in the Multidisciplinary Oncology Group at your institution?**

- ☐ Yes
- ☐ No

**For the treatment of paragangliomas and pheochromocytomas, have you ever referred a case to an ENETS reference center or requested a teleconsultation from another institution?**

- ☐ No
- ☐ Yes

**In the pathological definition of PPGL, the histological report generally includes the following information:**

- ☐ Ki-67 index, S-100 protein, GATA3, SDHB immunohistochemistry, and other markers (SDHA, MAX, 2SC)
- ☐ Ki-67 index, S-100 protein, GATA3, SDHB immunohistochemistry, other markers (SDHA, MAX, 2SC), and PSS-score
- ☐ Ki-67 index, S-100 protein, GATA3, SDHB immunohistochemistry, other markers (SDHA, MAX, 2SC), PSS-score, and GAPP-score
- ☐ I do not know
- ☐ Other (please specify)

**At the time of diagnosis, is the patient referred for oncogenetic counseling?**

- ☐ No
- ☐ Yes, for the investigation of Von Hippel-Lindau (VHL) disease caused by VHL mutations
- ☐ Yes, for the investigation of MEN2 caused by RET mutations
- ☐ Yes, for the investigation of VHL, MEN2, and familial PPGL
- ☐ Other (please specify)

**Which functional imaging modality do you request in addition to conventional radiology for the diagnosis of PPGL?**

- ☐ 123I-meta-iodobenzylguanidine (123I-MIBG) scintigraphy
- ☐ 68Ga-DOTA-peptide PET
- ☐ 18F-DOPA PET
- ☐ 18F-FDG PET/CT
- ☐ Other (please specify)

**For the treatment of metastatic cases, which first-line treatment do you primarily consider?**

- ☐ SSA-LAR (if 68Ga-DOTA-peptide PET-positive)
- ☐ Chemotherapy with cyclophosphamide + vincristine + dacarbazine (CVD)
- ☐ Enrollment in clinical trials (please specify)
- ☐ Other (please specify)

**In the last 10 years, how many metastatic patients have you treated with chemotherapy (CVD regimen) as a first-line treatment?**

- ☐ 0
- ☐ 1-5
- ☐ 6-10
- ☐ 11-20
- ☐ >20

**In the last 10 years, how many metastatic patients have you treated with SSA-LAR as a first-line treatment?**

- ☐ 0
- ☐ 1-5
- ☐ 6-10
- ☐ 11-20
- ☐ >20

**In the last 10 years, how many metastatic patients have you treated with Radio Ligand Therapy (RLT) as a first-line treatment?**

- ☐ 0
- ☐ 1-5
- ☐ 6-10
- ☐ 11-20
- ☐ >20

**In the last 10 years, how many metastatic patients have you treated with Tyrosine Kinase Inhibitors (TKIs) as a first-line treatment?**

- ☐ 0
- ☐ 1-5 (specify the type of treatment)
- ☐ 6-10 (specify the type of treatment)
- ☐ 11-20 (specify the type of treatment)
- ☐ >20 (specify the type of treatment)
- ☐ Other (please specify)

**If applicable, please specify the type of TKIs treatment:**
*(Free text field)*

**In the last 10 years, how many metastatic patients have you treated with enrollment in clinical trials as a first-line treatment?**

- ☐ 0
- ☐ 1-5
- ☐ 6-10
- ☐ 11-20
- ☐ >20

**If applicable, please specify the type of clinical trial:**
*(Free text field)*

**In the last 10 years, how many metastatic patients have you treated with 'other' as a first-line treatment?**

- ☐ 0
- ☐ 1-5 (specify the type of treatment)
- ☐ 6-10 (specify the type of treatment)
- ☐ 11-20 (specify the type of treatment)
- ☐ >20 (specify the type of treatment)

**If applicable, please specify the type of treatment:**
*(Free text field)*

**Are there any clinical trials at your institution enrolling patients with pheochromocytoma and paraganglioma?**

- ☐ Yes
- ☐ No

**If applicable, please specify the type of trial:**
*(Free text field)*

**Regarding second-line treatment for metastatic patients, how many have you treated with chemotherapy in the last 10 years?**

- ☐ 0
- ☐ 1-5 (please specify the treatment regimen)
- ☐ 6-10 (please specify the treatment regimen)
- ☐ 11-20 (please specify the treatment regimen)
- ☐ >20 (please specify the treatment regimen)

**If applicable, please specify the treatment regimen:**
*(Free text field)*

**Regarding second-line treatment for metastatic patients, how many patients have you treated with SSA-LAR in the last 10 years?**

- ☐ 0
- ☐ 1-5
- ☐ 6-10
- ☐ 11-20
- ☐ >20

### **Regarding second-line treatment for metastatic patients, how many patients have you treated with RLT in the last 10 years?**

- ☐ 0
- ☐ 1-5
- ☐ 6-10
- ☐ 11-20
- ☐ >20

### **Regarding second-line treatment for metastatic patients, how many patients have you treated with TKIs in the last 10 years?**

- ☐ 0
- ☐ 1-5 (specify the treatment regimen)
- ☐ 6-10 (specify the treatment regimen)
- ☐ 11-20 (specify the treatment regimen)
- ☐ >20 (specify the treatment regimen)

**If applicable, please specify the type of TKIs:**
*(Free text field)*

### **Regarding second-line treatment for metastatic patients, how many patients have you enrolled in clinical trials in the last 10 years?**

- ☐ 0
- ☐ 1-5
- ☐ 6-10
- ☐ 11-20
- ☐ >20

**If applicable, specify the type of clinical trial:**
*(Free text field)*

### **Regarding second-line treatment for metastatic patients, how many patients have you treated with "other" in the last 10 years?**

- ☐ 0
- ☐ 1-5 (specify the type of treatment)
- ☐ 6-10 (specify the type of treatment)
- ☐ 11-20 (specify the type of treatment)
- ☐ >20 (specify the type of treatment)

**If applicable, specify the type of treatment:**
*(Free text field)*

**Have you ever treated/referred patients with pheochromocytoma or paraganglioma for RLT? If yes, specify the radiopharmaceutical used (131I-MIBG, 90Y-DOTA-peptide, 177Lu-DOTA-peptide).**

- ☐ Yes
- ☐ No

**If applicable, specify the type of radiopharmaceutical used:**
*(Free text field)*

**Based on your experience, which treatment has resulted in the highest objective response rate?**

- ☐ SSA-LAR (if 68Ga-DOTA-peptide PET-positive)
- ☐ Chemotherapy with CVD
- ☐ Radio Ligand Therapy (RLT)
- ☐ Enrollment in clinical trials
- ☐ I do not know
- ☐ Other (please specify)

**Based on your experience, which treatment has provided the longest duration of response?**

- ☐ SSA-LAR (if 68Ga-DOTA-peptide PET-positive)
- ☐ Chemotherapy with CVD
- ☐ Radio Ligand Therapy (RLT)
- ☐ Enrollment in clinical trials
- ☐ I do not know
- ☐ Other (please specify)

**Based on your experience, which treatment has provided the best quality of life?**

- ☐ SSA-LAR (if 68Ga-DOTA-peptide PET-positive)
- ☐ Chemotherapy with CVD
- ☐ Radio Ligand Therapy (RLT)
- ☐ Enrollment in clinical trials
- ☐ I do not know
- ☐ Other (please specify)
